# Three-Year-Olds Solved a Mental Rotation Task Above Chance Level, but No Linear Relation Concerning Reaction Time and Angular Disparity Presented Itself

**DOI:** 10.3389/fpsyg.2018.01796

**Published:** 2018-10-04

**Authors:** Markus Krüger

**Affiliations:** Entwicklungspsychologie und Pädagogische Psychologie, Institut für Psychologie, Universität Greifswald, Greifswald, Germany

**Keywords:** mental rotation, imagery, mental transformations, mental representations, spatial cognition, infants, early competencies, habituation

## Abstract

Three-year-olds and 4-year-olds have severe difficulties solving standard mental rotation tasks. Only 5-year-olds solve such tasks above chance reliably. In contrast studies relying on simplified mental rotation tasks indicate that infants discriminate between an object and its mirror image. Furthermore in another simplified mental rotation task with 3-year-olds, a linear relation between angular disparity and reaction time typical for mental rotation was revealed. Therefore it was assumed that 3-year-olds’ capabilities are underestimated. In the current study, 3-year-olds were trained in two isolated sessions to solve standard mental rotation tasks and were tested in a third session. Three-year-olds solved this test above chance as a group – a substantial number of them doing so on an individual level. However, a linear relation between angular disparity and reaction time, that would indicate an analog mental transformation, was not discernable. Nevertheless, these findings are in accordance with a continuous line describing mental rotation in infants and older children. And, these also indicate that children’s mental rotation capabilities might be underestimated.

## Introduction

Mental rotation is a special case of an analog mental transformation (Shepard and Metzler, 1971). Participants are presented with two pictures that either depict the same object from different perspectives or an object and its mirror image, again from different perspectives. The time participants need to decide whether they see the same or the converse objects corresponds with the angular disparity between the depicted objects. Greater angular disparities lead to longer reaction times (RTs). Typically, this results in a linear relationship between angular disparity and RT. The accepted explanation for this phenomenon is that an analog mental transformation takes place: Participants rotate their representation of one object about the shortest path until it matches the other. This takes longer the more rotation is required.

These analog mental transformations might be an inherent human feature. Therefore it is not surprising that infants’ ability to differentiate between objects and their mirror images is interpreted as mental rotation (e.g., Moore and Johnson, 2008; Quinn and Liben, 2008; Schwarzer et al., 2013). Usually, mental rotation paradigms for infants follow the same structure (see Mash et al., 2008): Infants are habituated to a geometric object that rotates back and forth. Then, they are confronted with either the same object or its mirror image, both from a new perspective, again rotating. As infants tend to dishabituate when presented with the mirror image but not when presented with the same object it is concluded that they detect the difference.

Infants’ early competence is in contrast to toddlers’ and even kindergartners’ performance in mental rotation tasks. Generally, 3-year-olds fail completely in standard mental rotation tasks and only a minority of 4-year-olds shows signs of mental rotation, while 5-year-olds solve mental rotation tasks at group level (i.e., mean performance is above chance) reliably (e.g., Estes, 1998; see Frick et al., 2014, for an overview; but, see Marmor, 1977, for mental rotation in 4-year-olds). In a recent study, Frick et al. (2013) asked participants to decide in which of two holes a puzzle piece would fit. The holes were mirror images of each other and puzzle pieces were presented in different rotations. While 5-year-olds could solve this task above chance on a group level, 4-year-olds could not do so.

Such discrepancies between infants’ competence and children’s perceived incompetence are not uncommon. For example, while infants infer hidden objects when these would explain an otherwise physically impossible event (Baillargeon, 2004), 3-year-olds and even older children fail to do so (Krist et al., 2016). Similar discrepancies cannot only be found in intuitive physics alone, but also in social cognition (see Bian and Baillargeon, 2017). It might seem implausible that toddlers and older children perform very poorly in tasks that require competencies infants already have. A prominent explanation for this phenomenon is a representational redescription that reshapes former competencies or turns them inaccessible (see Carey, 2009). Furthermore, these discrepancies are often attributed to different task demands (see Keen, 2003; e.g., Aschersleben et al., 2013). According to this approach, children do not fail because they lack the (from a theoretical point of view) critical competence, but are overwhelmed by the demands resulting from other aspects of the tasks. We can only speculate on the task demands for the infant mental rotations tasks. It seems that participants require a representation of the object shown during habituation lasting at least to the beginning of the test phase. Furthermore, if an analog mental transformation of the objects takes place it might be externally supported by the shown rotation (see timing-responsive representations, Schwartz and Holton, 2000; cf. Krüger and Krist, 2017).

There is empirical evidence, that in the case of the mental rotation paradigm, task demands might play a decisive role. In a recent study, children aged from 3 to 6 years were tested with a simplified mental rotation task with reduced task demands (Krüger et al., 2014). It turned out that even the tested 3-year-olds were able to solve this task. As in classical mental rotation, two objects were presented – one upright and one rotated. However, there were no mirror images nor had the children to decide, whether the objects were the same or different. Instead they were asked to bring the rotated object into an upright position by rotating it along the shortest path. It was measured how much time children needed to start the manual rotation (RT), because a mental transformation to determine the shortest path preceding the manual rotation was assumed. It turned out that children of all tested age groups were able to solve this task reliably (i.e., find the shortest path) at group level. About half of the 3-year-olds were above chance on an individual level. Moreover, as RTs rose linearly with the angular disparity between the presented objects, it was concluded that participants used analog mental transformations to solve this task.

Of course, in that paradigm (Krüger et al., 2014) task demands were reduced: The participants were spared the necessity to represent two objects at the same time to compare them. And they did not have to make a decision whether the objects were identical, nor was there the need to express this decision. In this reduced task, once the shortest path was established by the analog mental transformation, there was no need to maintain the mental representation any further. One might argue, that high task demands are one of the reasons why younger children fail classical mental rotation tasks (see Frick et al., 2014).

It is even more astonishing that the ability to differentiate between an object and its mirror image is a feat that infants seem to accomplish (e.g., Mash et al., 2008). Therefore, the goal of the present study was to test, whether 3-year-olds could accomplish this, too. This would further close the gap between infants and kindergartners by including this determining aspect of the infants’ mental rotation in the 3-year-olds’ task.

The test for the 3-year-olds was designed as closely as possible to the original mental rotation test (Shepard and Metzler, 1971). There were only two concessions: (1) Instead of quasi 3D stimuli, 2D stimuli were used (which is common for testing mental rotation in children since Marmor, 1975). (2) Instead of two stimuli three stimuli were presented: one large central picture and two smaller comparison pictures. Participants had to decide which of the smaller pictures matched the central one. Such configurations have been used before in order to test adults (e.g., Wohlschläger and Wohlschläger, 1998) and children (e.g., Krüger and Krist, 2009).

The task demands were exactly the same as in other studies with older children. The new aspect of the current study was not to reduce task demands directly by simplifying the task, but to reduce them indirectly by furthering automatization of basal processes through training (see Paas et al., 2004). To test mental rotation in very young children, extensive training was carried out before – either by practicing the task itself or by getting participants acquainted with the stimulus material through manual exploration, etc. (e.g., Frick et al., 2013). For the current study, it was assumed that an effective training must allow for the automatization and in turn time for consolidation was needed (e.g., Wilhelm et al., 2012). Therefore, multiple training sessions on different days were implemented (see also, Marmor, 1975, 1977). Furthermore, during the training sessions children were given the opportunity to manually rotate the central stimulus by using a touchscreen (see also, Krüger et al., 2014) and an explicit imagery instruction was given.

## Materials and Methods

### Participants

A total of 60 3-year-olds were recruited for this study, however, only 42 of them were present at all three sessions (18 children were excluded, because they did not show up often enough in their kindergarten to participate in all three sessions). One child was older than 3 years when the last session took place and was excluded from final data analysis. Two children were younger than 3 years when the first session took place and were included. Of the remaining 41 children (age at the first session: *M* = 40 months, *SD* = 3, min = 35, and max = 45), 15 were boys and 26 were girls.

All the children were tested in a separate room in their kindergarten by the same female experimenter. All the kindergartens were located in Berlin, Germany. All parents were informed about the goal and procedure of the study. They had the opportunity to ask for clarification. Children participated with the written consent of their parents, but even after consent was given, children could end cooperation anytime on their own behalf. Participation was rewarded with sweets and coloring pictures. No approval by an ethics committee was required for this study by our institution. This study was conducted in accordance with the ethical guidelines of the German Psychological Society [Deutsche Gemeinschaft für Psychologie (DGPs)].

### Materials

Test stimuli consisted of 12 hand drawn and then digitalized pictures of 2D animate and inanimate objects (**Appendix A**) and their mirror images. Pictures were asymmetric to allow participants to differentiate between the pictures and the corresponding mirror images. All pictures and their mirror images were rotated from their baseline (0°) by 45°, 90°, 135°, 165°, 195°, 225°, 270°, and 315°. The 165° and 195° were chosen instead of the usual 180° to offer an unambiguous shortest path for rotation.

All stimuli were presented on a Clevo Co., eTouch TN12T notebook (12′′, 1280 pixels × 800 pixels) with a touchscreen. E-Prime software was used for presentation and measurement.

### Procedure

**FIGURE 1:**
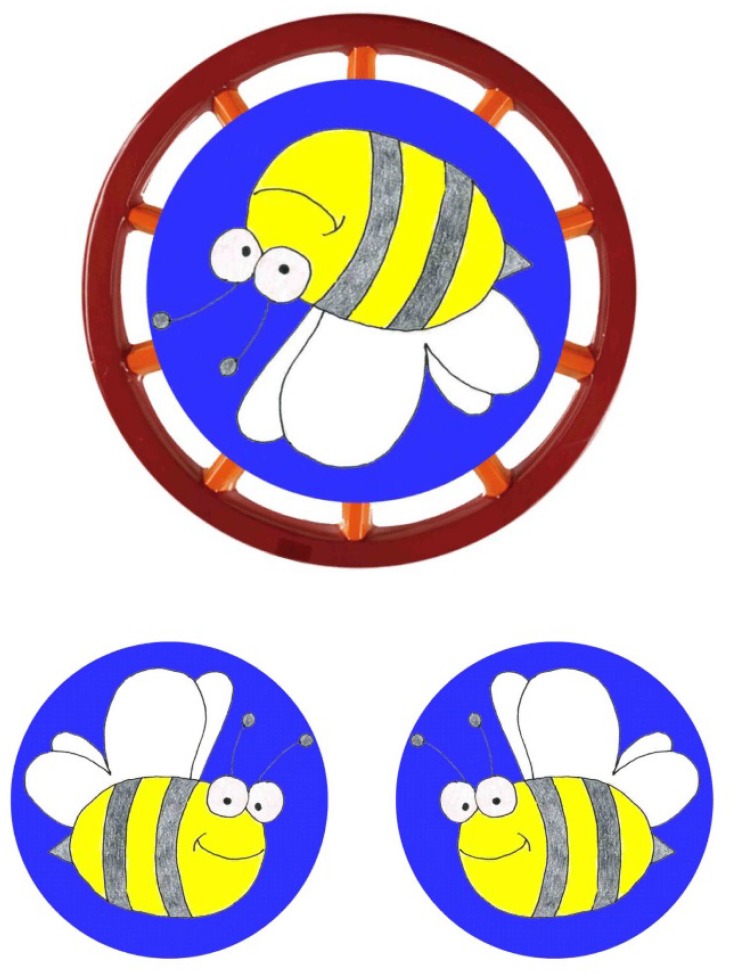
Example of the stimulus configuration as presented on the screen (here: bee at 195°).

Participants were always presented with a central picture and two smaller comparison pictures below (**Figure 1**). The children’s task was to touch the small picture that corresponded with the central picture (the software recorded automatically which picture was touched and the moment it happened). When they touched the correct picture a smiley appeared on the screen and a pleasant tune was played; when the wrong picture was touched a frowny appeared and an unpleasant tune was played. Every trial was triggered by the experimenter by pressing a button. The experimenter ensured that children placed their hands on a mat in front of the monitor and waited until children looked at the monitor before releasing each trial. RT was measured from stimulus onset on (i.e., when the stimulus configuration as seen in **Figure 1** appeared).

Usually, children participated in the sessions weekly, with a break of 7–8 days between sessions.

Details differed between the 3 sessions (2 training sessions and 1 final test session) as described below.

#### First Session

The first session was a training session consisting of 49 trials. The central picture was fitted with a “handle” and it was rotatable. The first trial was always the fish and the central picture rotated about 135°. The experimenter demonstrated how the central picture could be manually rotated by dragging the handle on the touchscreen. Then, she rotated the central fish into the 0° position. She told the participants, that in this upright position it was easy to see which of the comparison pictures corresponded with the central picture. After that she studied both comparison pictures and touched the matching picture. After this demonstration, 48 trials (3 [objects: chicken, bee, and moon] × 8 rotations [45°, 90°, 135°, 165°, 195°, 225°, 270°, and 315°] × 2 laterality [corresponding comparison picture left or right]) followed in a random order. The children were encouraged to solve the task on their own.^1^ However, the experimenter answered all their questions and repeated the instructions if necessary. When children stopped using the rotation of the central picture to solve the task on their own, the experimenter did not comment on their choice or enforce the use of the manual rotation.

#### Second Session

The second session consisted of 68 trials. As in the first session, the central picture was fitted with a handle and was rotatable. The first four trials were predetermined (chicken at 45°, chicken at 225°, bee at 165°, and bee at 315°). Then 64 trials followed (4 [objects: caterpillar, snail, seagull, and teapot] × 8 rotations [45°, 90°, 135°, 165°, 195°, 225°, 270°, and 315°] × 2 laterality [corresponding comparison picture left or right]) in a random order. Again, children were asked to solve the task, as they had learned. After the first four trials, children were asked to continue without manually rotating the central picture, but just to imagine to do so (imagery instruction). However, the use of the handle was still allowed. As in the first session, the experimenter answered all questions and repeated the instructions if necessary. If children continued to use the handle, they were encouraged to manage without it.

#### Third Session

The third session consisted of 64 trials (4 [objects: watering can, car, boat, and duck] × 8 rotations [45°, 90°, 135°, 165°, 195°, 225°, 270°, and 315°] × 2 laterality [corresponding comparison picture left or right]) presented in a random order. None of the objects had been presented in the sessions before. There was no handle and the central picture was not rotatable. Before the trials started, children were informed that they had to solve the task without any manual rotation. The experimenter did not answer any further questions.

## Results

The focus of this study was, whether children were able to solve this mental rotation task. This was tested in the third session. Therefore, all data reported in the results section refer to the third session.

### Accuracy

**FIGURE 2:**
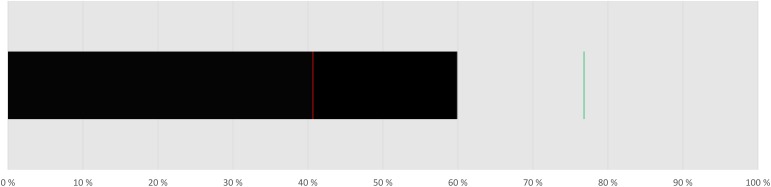
Medium ACC as hit rate in percent. The red line indicates the lowest and the green line the highest individual hit rate.

On a group level, children’s performance was better than chance (*M* = 38.22 hits, *SD* = 5.45, min = 26, max = 49, and hit rate = 59.7 %), *t*(40) = 7.31, *p* < 0.001, and *d*_z_ = 1.16 (see Lakens, 2013), indicating that participants were not simply guessing (**Figure 2**). Moreover, the number of individuals that were above chance [40 hits or better out of 64 (hit rate = 62.5%)] according to a binomial distribution, (*p* < 0.05) on an individual level was counted. Sixteen individuals reached this criterion. These results indicate that 3-year-olds are able to solve classical mental rotation tasks.

No difference between the performance of boys (*M* = 38.0 hits, *SD* = 5.24, and hit rate = 59.4%) and girls (*M* = 38.35 hits, *SD* = 5.66, and hit rate = 59.9%) was detectable, *p* > 0.20. There was no indication that the performance differed due to angular disparity, *F* < 1 (**Table 1**) or the four different test stimuli, *p* > 0.20.

**Table 1:** ACC (and *SD*) and hit rates for the different angles of rotation.

Angle	045°	090°	135°	165°	195°	225°	270°	315°
ACC	*M* = 4.7	*M* = 5.0	*M* = 4.6	*M* = 4.8	*M* = 4.6	*M* = 5.0	*M* = 4.4	*M* = 5.1
	*SD* = 1.4	*SD* = 1.4	*SD* = 1.6	*SD* = 1.1	*SD* = 1.5	*SD* = 1.3	*SD* = 1.5	*SD* = 1.4
	58.8%	62.5%	57.5%	60.0%	57.5%	62.5%	55.0%	63.8%

*There were eight possible hits for each angle*.

### Reaction Times

For analysis, only RTs of trials with correct solutions were considered, RTs that were smaller than 1 *SD* or larger than 2 *SD* than the group mean were excluded (**Supplementary Appendix B**), and RTs that had the same angular disparity for the shortest rotation path were pooled (e.g., 90° and 270°; see Shepard and Metzler, 1971), resulting in four different rotation angles. Mean RT was *M* = 4196 ms, *SD* = 1543 (**Supplementary Appendix C**).

A 4 (angles: 45°, 90°, 135°, and 165°) × 2 (sex) ANOVA was computed. There was no effect for the factor angle, *F* < 1, or discernable trends – linear or otherwise, all *F*s < 1. Even descriptively, there was no indication of RTs getting longer with higher angular disparity. There were no other effects or interactions, all *F*s < 1. This analysis was repeated for those 16 children that were above chance on an individual basis with the same results.

### Rotation Direction

In the first session, children used the handle to manually rotate the central stimulus. The rotation direction was recorded for each trial. An additional analysis was conducted to test whether children had a preferred rotation direction that might suppress the expected linear trend. Therefore, a clockwise rotation was counted as +1, a counter-clockwise rotation as -1, and no rotation as zero. For the first session, this led to a directional score from -48 to +48. A large negative score indicated a tendency for counter-clockwise rotation and a high score indicated a tendency for clockwise rotation. However, no substantial tendency for one or the other rotation direction was discernable, *M* = 2.68, *SD* = 13.81, min = -29, max = 42, and *p* > 0.20.

Nevertheless, the relatively large standard deviation and the distinctive maximum and minimum justify the thought that there might be individuals that had a directional preference. These individuals might have taken this preference to the second session and to the third session. Therefore, the three participants with the highest and the three participants with the lowest directional score were sheared off and the 4 (angles: 45°, 90°, 135°, and 165°) × 2 (sex) ANOVA was computed without them. As before, this yielded no significant results, all *F*s < 1, indicating that a possible linear trend was not due to single individuals with a preferred rotation direction.

## Discussion

On the one hand, as a group the 3-year-olds demonstrated their ability to solve a mental rotation task clearly above chance level. There was also a number of 3-year-olds who performed better than chance on an individual level. On the other hand there was no indication of the linear trend typical for mental rotation.

Compared with infant studies, these results should not be surprising. When we accept that infants do differentiate between objects and their mirror images, it is reasonable to expect that 3-year-olds can do so, too. But infants’ performance is difficult to quantify with the current habituation paradigms and defies a direct comparison with the 3-year-olds’ performance in a classical mental rotation task. With the current habituation paradigms performance can only be assessed at a group level by comparing looking-times (see Mash et al., 2008). It is not possible to reliably identify individual above chance performers or to quantify the number of trials that were solved correctly. However, hit rates for children older than the ones tested by us exist. For instance, in their puzzle mental rotation paradigm Frick et al. (2013) found that 4-year-olds performed below chance with a hit rate of 53.8% and 5-year-olds performed reliably above chance level with a hit rate of 67.5%, putting the 3-year-olds tested here between these age groups with 59.7%.

This puts our current results at odds with recent research (see Frick et al., 2014), but it seems to fit better when taking a closer look at earlier research. When pioneering mental rotation research in children, Marmor (1975, 1977) trained participants over a stretch of 4 days and found that 4-year-olds could indeed solve mental rotation tasks. This finding indicates that the failure of younger children in mental rotation tasks might be due to task demands and that this situation can be remedied by extended training (see Keen, 2003).^2^ Or, the other way round, it seems possible that recent research with children using mental rotations is too hasty (e.g., for our other mental rotation studies with children, 20–30 min are scheduled per participant). Thus, children’s abilities are underestimated.

Nevertheless, it is especially noteworthy that the linear relationship between angular disparity and RTs was not found in our current study. This was even so when looking at the high performing participants only or when considering that some participants might have rotated mostly in the same direction instead of choosing the shortest path. It is unlikely that this is caused by the used mental rotation paradigm (central picture with comparison pictures), as this paradigm was used with adults and older children before and always yielded a linear trend (e.g., Wohlschläger and Wohlschläger, 1998; Krüger and Krist, 2009). It might be possible that performing on the edge of their capabilities (see Frick et al., 2014) has blurred the finer details of 3-year-olds’ mental rotation processes. Again previous research with older children is heterogeneous. While Frick et al. (2013) did not find a linear connection between RT and angular disparity in 4-year-olds, Marmor (1977) did. As for infants, the current habituation paradigms do not allow for the meaningful measurement of RT, as there is no explicit time point when infants “reply” (see Mash et al., 2008).

Of course, such a linear trend would be highly informative, as it would have established how our participants solved the task, namely by analog mental transformations (Shepard and Metzler, 1971). In turn, the apparent lack of such a connection between RTs and angular disparity casts doubt on the assumption that what we observed here in the 3-year-olds is the same as mental rotation in older children and adults. Therefore, a clear indication for the way the 3-year-olds solved the task is missing. This problem is valid for infants, too. Nevertheless, there is, as described in the Introduction, evidence from another study, that 3-year-olds use analog mental transformations when solving a different, simplified mental rotation task (Krüger et al., 2014). And, there are indications that adults process the infant mental rotation tasks in a quite similar way as infants. Heil et al. (2018) found a high correlation between performance in an adapted infant task (Moore and Johnson, 2008) and performance in a mental rotation test (Peters et al., 1995) in an adult sample. Therefore, it seems conceivable that participants used analog mental transformations in the present task and in the infant tasks, also.

## Conclusion

Especially the missing conformation of a linear connection between angular disparity and RTs makes the interpretation of the present findings ambiguous. In principle there are two very different conclusions possible.

On the one hand one can – in line with Lev Vygotsky – argue that with enough coaching and patience it is possible to reveal abilities in children that are not visible at a cursory glance. The phenomenon we have observed here is indeed mental rotation based on analog mental transformations in 3-year-olds. Also, the same might be true for infants’ ability to differentiate between an object and its mirror-image in simplified mental rotation tasks. Thus, the ability to use analog mental transformations is inherent from an early age on and there is no gap between seemingly rotating infants and clearly rotating 5-year-olds. Such an interpretation bears the risk of overestimating children’s cognition.

On the other hand one can follow a more strict interpretation – comparable to Jean Piaget’s approach. Thus, 3-year-olds are able to solve classical mental rotation tasks only after it was revealed to them how to do it and even then there was no direct evidence for analog mental transformations being involved. So it remains unclear, how exactly they managed to be substantially more often right than wrong. Habituation studies provide no evidence of any conceptual understanding of mental rotation, let alone analog mental transformations. And, it is questionable that looking longer at one object than at another or performing a task better than chance, but far from perfect, should be seen as competence. Of course, such an interpretation bears the risk of underestimating children’s cognition.

## Author Contributions

The author confirms being the sole contributor of this work and approved it for publication.

## Conflict of Interest Statement

The author declares that the research was conducted in the absence of any commercial or financial relationships that could be construed as a potential conflict of interest.

## Figures and Tables

**FIGURE A1:**
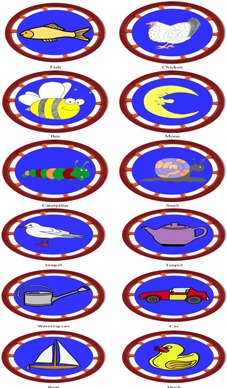
Display of all stimuli fitted with the “handle”.

## Footnotes

1 In some mental rotation studies participants are asked to answer as quickly and correctly as possible. That was not the case here. According to the signal detection theory this seems to be a paradox demand. Adult participants usually interpret this demand to find an equilibrium between both optimizations, but it seems doubtful that young children understand the necessary comparatives. Nevertheless, this might influence the results – especially the RTs. However, in a simplified mental rotation task with the same age group and without such an instruction, RTs were typical for mental rotation (Krüger et al., 2014, Exp. 1).

2 However, it must be noted that children’s performance was not necessarily due to task demands. It might as well be that the training helped children to understand what the task required them to do (see Marmor, 1975). A combination of both approaches is also possible.
