# Supplementary material for: Three-Year-Olds Solved a Mental Rotation Task Above Chance Level, but No Linear Relation Concerning Reaction Time and Angular Disparity Presented Itself
Source: Front Psychol. 2018 Oct 4;9:1796. doi: 10.3389/fpsyg.2018.01796 (PMC6180201; doi:10.3389/fpsyg.2018.01796)
Supplement: Supplementary file 1 [file Data_Sheet_1.pdf]

Appendix B

For the whole sample (*N* = 42) 122 RT measurements were identified as outliers (1 *SD* smaller or 2 *SD* larger than the group mean).

Angle	045°	090°	135°	165°	195°	225°	270°	315°
Outliers	16	16	11	20	16	11	14	18

Appendix C

RT (and *SD*) of the 16 children performing above chance level for the different angles of rotation.

Angle	045°	090°	135°	165°	195°	225°	270°	315°
RTs in ms	*M* = 4356<br><br>*SD* = 2152	*M* = 3653<br><br>*SD* = 1318	*M* = 3649<br><br>*SD* = 1886	*M* = 4378<br><br>*SD* = 1892	*M* = 3859<br><br>*SD* = 1254	*M* = 3947<br><br>*SD* = 1239	*M* = 4500<br><br>*SD* = 1867	*M* = 3388<br><br>*SD* = 1485
